# Treatment variation in stent choice in patients with stable or unstable coronary artery disease

**DOI:** 10.1007/s12471-015-0783-5

**Published:** 2016-01-13

**Authors:** L. T. Burgers, E. A. McClellan, I. E. Hoefer, G. Pasterkamp, J. W. Jukema, S. Horsman, N. H. J. Pijls, J. Waltenberger, M. A. Hillaert, A. C. Stubbs, J. L. Severens, W. K. Redekop

**Affiliations:** 1 Institute of Health Policy & Management, and Institute for Medical Technology Assessment, Erasmus University Rotterdam, Rotterdam, The Netherlands; 2 Department of Mathematical and Computer Sciences, Metropolitan State University of Denver, Colorado, USA; 3 Laboratory of Experimental Cardiology, UMC Utrecht, Utrecht, The Netherlands; 4 Department of Cardiology, Leiden UMC, Leiden, The Netherlands; 5 Department of Bioinformatics, Erasmus Medical Center, Rotterdam, The Netherlands; 6 Department of Cardiology, Catharina Hospital Eindhoven, Eindhoven, The Netherlands; 7 Department of Cardiology, Maastricht University Medical Center, Maastricht, The Netherlands; 8 Department of Cardiovascular Medicine, University of Münster, Münster, Germany; 9 Department of Cardiology, University Medical Centre Utrecht, Utrecht, The Netherlands

**Keywords:** Treatment variation, Coronary artery disease, Drug-eluting stent, Bare-metal stent, Percutaneous coronary intervention

## Abstract

**Aim:**

Variations in treatment are the result of differences in demographic and clinical factors (e.g. anatomy), but physician and hospital factors may also contribute to treatment variation. The choice of treatment is considered important since it could lead to differences in long-term outcomes. This study explores the associations with stent choice: i.e. drug-eluting stent (DES) versus bare-metal stents (BMS) for Dutch patients diagnosed with stable or unstable coronary artery disease (CAD).

**Methods & results:**

Associations with treatment decisions were based on a prospective cohort of 692 patients with stable or unstable CAD. Of those patients, 442 patients were treated with BMS or DES. Multiple logistic regression analyses were performed to identify variables associated with stent choice. Bivariate analyses showed that NYHA class, number of diseased vessels, previous percutaneous coronary intervention, smoking, diabetes, and the treating hospital were associated with stent type. After correcting for other associations the treating hospital remained significantly associated with stent type in the stable CAD population.

**Conclusions:**

This study showed that several factors were associated with stent choice. While patients generally appear to receive the most optimal stent given their clinical characteristics, stent choice seems partially determined by the treating hospital, which may lead to differences in long-term outcomes.

## Introduction

Despite improvement in the prognosis of patients with cardiovascular disease (CVD) it still remains the second leading cause of death across the Western world and one of the major causes of disability [1]. For many years patients with coronary artery disease (CAD), the most frequent type of CVD, were treated mainly with percutaneous coronary intervention (PCI), coronary artery bypass grafting (CABG) or medication only. Both revascularisations reduce the incidence of death and myocardial infarction (MI) in CAD patients compared with no treatment, but most patients are now treated with PCI. In 2012 approximately 39,000 PCIs were performed in the Netherlands [2]. Originally a PCI was performed with an expanding balloon; however, nowadays patients are often treated with a bare-metal stent (BMS) or drug-eluting stent (DES). DES reduces restenosis compared with BMS (8.4 versus 20.9 %) [3]. However, patients treated with DES, especially the early-generation, might have a higher chance of developing very late stent thrombosis (0.7 versus 0.1 %) [3]. Both types of stents have pros and cons; decisions should be based on what is considered appropriate for a patient since the choice of stent type may have impact on long-term outcomes. Variations in treatment are the result of differences in patient characteristics and clinical factors (e.g. anatomy) but previous studies have shown that physician and hospital factors may contribute to treatment variation. In the UK, stent choice was associated with the operator and the treating hospital [4]. Tu et al. [5] have shown that the physician performing the diagnostic catheterisation and the treating hospital were strong independent predictors of the type of revascularisation (CABG versus PCI) in Canada. Furthermore, the type of stent was also determined by the type of payer (e.g. Medicaid, private insurance) [6]. Of course, these results may be expected to be healthcare system specific and do not apply for Dutch patients, since the Netherlands has a centrally publicly funded healthcare system.

This study will explore the associations with stent choice (DES or BMS) for Dutch patients diagnosed with stable or unstable CAD focusing on variation due to clinical factors and treating hospital.

## Methods

### Study design

Treatment variation of patients with stable or unstable CAD was explored through analysing data from the Circulating Cells prospective cohort study, which has the aim of discovering markers that identify patients who are at an increased risk of developing a cardiovascular event. In this multicentre study, patients undergoing coronary angiography were included if they had known or suspected stable or unstable CAD; specific diagnoses included unstable angina and non-ST-elevation MI (NSTEMI) [7].

### Treatment

Patients undergoing coronary angiography were asked to participate in the study. Data were collected regarding patient characteristics, test results and treatment decisions. Patients who were treated with a PCI received a BMS, DES, drug-eluting balloon angioplasty or standard balloon angioplasty. The aim of this study is to examine the factors that are associated with stent choice (DES vs. BMS), meaning that patients treated solely with drug-eluting balloon angioplasty or standard balloon angioplasty are excluded from the analyses. Stent choice for DES was defined as a PCI with at least one DES, including patients treated with only DES but also patients treated with DES in combination with BMS, drug-eluting balloon angioplasty or standard balloon angioplasty. Stent choice for BMS was defined as a PCI with only BMS such that patients treated with BMS in combination with balloon angioplasty or DES are excluded.

### Data and statistical analyses

Choice of stent type (DES or BMS) was compared between patient subgroups, determined by diagnosis. The following baseline characteristics were also collected during the study: age, gender, body mass index (BMI), systolic blood pressure (SBP), diastolic blood pressure (DBP), thrombolysis in myocardial infarction (TIMI) score for unstable CAD patients, New York Heart Association (NYHA) class, number of diseased vessels (50–99 % stenosis), cardiac history (previous heart failure, previous MI, previous PCI, and previous CABG), non-cardiac history (cerebrovascular accident (CVA) or transient ischaemic attack (TIA), pulmonary disease, peripheral vessel disease (PVD), and renal failure), and CVD risk factors (diabetes mellitus, hypertension, hyperlipidaemia, smoking, and pack-years (tobacco)).

Multiple imputation was used to prevent patients from being excluded from the analyses due to missing values. Baseline characteristics (SBP, DBP, BMI, NYHA class, previous heart failure, previous MI, CVA or TIA, pulmonary disease, PVD, renal failure, diabetes mellitus, hypertension, hyperlipidaemia, current smoker and pack-years) were missing for less than 2 % of all cases except pack-years, which was missing for 14 % of all cases. These characteristics were imputed using predictive mean matching for scale variables. Five imputation sets were created with ten iterations, each using fully conditional specification in SPSS 22 (IBM Corp. Released 2013. IBM SPSS Statistics for Windows, Version 22.0. Armonk, NY: IBM Corp). Age, gender, previous PCI, previous CABG, and diagnosis were only used as predictors and not imputed since there were no missing values for these variables.

Differences between groups were tested using Chi-squared analysis for categorical variables. Bivariate analyses using logistic regression were performed to identify variables that were associated with stent type; stable and unstable CAD patients were analysed separately. Backwards selection was used to create the final multivariate model(s). P values lower than 0.05 were considered statistically significant, although a higher threshold of 0.1 was used to select variables for the multivariate analysis. Associations were discussed with clinical experts in order to see if the results make sense (face validity).

## Results

In total, 714 patients were included in the Circulating Cells cohort, 22 of whom were excluded from the analyses since they did not have significant coronary atherosclerosis. The remaining 692 patients were included in three teaching hospitals and one general hospital, and 477 patients were treated with PCI. Of those patients, 442 patients were treated with BMS or DES. Others were treated with a combination of BMS and balloon angioplasty (*n* = 4), drug-eluting balloon angioplasty or standard balloon angioplasty (*n* = 18) or missing (*n* = 13) and are excluded from the analysis. The number of patients treated per hospital (I–IV) was 130, 98, 81, and 133, respectively. Table 1 presents the baseline demographic and angiographic characteristics of the included patients. The mean age of the cohort was 63 years and 72 % were male. The majority (81 %) of the patients were diagnosed with stable CAD (including silent ischaemia) after the coronary angiography. There were three significant differences in characteristics of stable CAD (*n* = 358) and unstable CAD patients (*n* = 84). Stable CAD patients more often had a lower NYHA class, were less often current smokers and had less often experienced a CVA/TIA compared with unstable CAD patients.

**Table 1:** Baseline characteristics

	All patients after imputation					Patients with stable CAD			Patients with unstable CAD			
	Mean		SD		*N* ^b^	Mean	SD	*N* ^b^	Mean	SD	*N* ^b^	*p* value^a^
*Baseline characteristics*												
Age		62.72		10	442	62.96	10	358	61.71	11	84	0.319
Male (%)		72 %			442	73 %		358	68 %		84	0.354
SBP (mmHg)		135		19	442	135	19	358	134	21	84	0.748
DBP (mmHg)		77		11	442	77	11	358	79	11	84	0.273
BMI (kg/m ^2^)		28		4	442	28	4	358	27	4	84	0.266
*TIMI score* ^c^												
1		8 %			83				8 %		83	
2		18 %			83				18 %		83	
3		30 %			83				30 %		83	
4		28 %			83				28 %		83	
5		12 %			83				12 %		83	
6 + 7		4 %			83				4 %		83	
Number of diseased vessels (50–99 %)												0.077
1		44 %			442	46 %		358	36 %		84	
> 1		56 %			442	54 %		358	64 %		84	
NYHA												***p*** **< 0.001**
NYHA I		73 %			442	73 %		358	76 %		84	
NYHA II		18 %			442	20 %		358	7 %		84	
NYHA III		6 %			442	7 %		358	4 %		84	
NYHA IV		2 %			442	0 %		358	13 %		84	
*Cardiac history (%)*												
Previous heart failure	2 %				442	2 %		358	1 %		84	0.542
Previous MI	31 %				442	33 %		358	23 %		84	0.066
Previous PTCA	33 %				442	35 %		358	26 %		84	0.116
Previous CABG	7 %				442	8 %		358	5 %		84	0.369
*Non-cardiac history (%)*												
CVA/TIA	8 %				442	6 %		358	14 %		84	**0.017**
Pulmonary disease	11 %				442	10 %		358	14 %		84	0.242
Peripheral vessel disease	13 %				442	13 %		358	14 %		84	0.684
Renal failure	3 %				442	4 %		358	1 %		84	0.25
*Risk factors (%)*												
Diabetes mellitus	21 %				442	22 %		358	20 %		84	0.764
Hypertension	66 %				442	67 %		358	60 %		84	0.189
Hyperlipidaemia	68 %				442	70 %		358	60 %		84	0.057
Current smokers	19 %				442	16 %		358	32 %		84	**0.001**
Pack years^d^	19.7		18		442	19.2	18.1	358	21.9	22.1	84	0.302
*Diagnosis (%)*												
Stable angina	81 %				442							
Unstable angina	10 %				442							
NSTEMI	9 %				442							
*Treatment/stent choice*												0.736
DES	66 %				442	66 %		358	68 %		84	
BMS	34 %				442	34 %		358	32 %		84	
*Hospital*												
I	29 %				442	28 %		358	37 %		84	
II	22 %				442	24 %		358	13 %		84	
III	18 %				442	14 %		358	37 %		84	
IV	30 %				442	34 %		358	13 %		84	

*BMI* body mass index, *CABG* coronary artery bypass graft, *CVA* cerebrovascular accident, *DBP* diastolic blood pressure, *MI* myocardial infarction, *NA* not applicable, *NSTEMI* non ST elevation myocardial infarction, *NYHA* New York heart association, *PTCA* percutaneous transluminal coronary angioplasty, *SBP* systolic blood pressure, *TIA* transient ischaemic attack, *TIMI* thrombolysis in myocardial infarction.

^a^Stable versus unstable.

^b^Number of patients on which the analyses were based.

^c^Only reported for unstable angina and NSTEMI.

^d^Number of packs per day multiplied with years of smoking.

In total 771 stents were used to treat 442 patients with 612 target lesions. On average 1.385 target lesions were stented per patient (range 1–3), where 1.260 stents were used per lesion and 1.744 stents (range 1–6) per patient were used. Of the 442 patients, 66 % were treated with one or more DES. Bivariate analyses (Table 2) showed that NYHA class, number of diseased vessels, previous PCI, smoking, diabetes and the treating hospital were significantly associated with stent choice for a patient. The frequency of DES use varied widely (50–99 %) between the four hospitals, considering the total population. The variation in stent choice was larger in the unstable patient group (45–100 %).

**Table 2:** Associations with therapeutic decision (DES vs BMS)

	All patients			Patients with stable CAD			Patients with unstable CAD		
	DES (%)/ OR	*N*	***p*** value	DES (%)/ OR	*N*	*p* value	DES (%)/ OR	*N*	*p* value
Overall	66 %	442		66 %	358		68 %	84	
Diagnosis			0.558						
Stable CAD	66 %	358							
Unstable angina	63 %	46							
NSTEMI	74 %	38							
Hospital			***p*** **< 0.001**			***p*** **< 0.001**			***p*** **< 0.001**
1	50 %	130		52 %	99		45 %	31	
2	64 %	98		66 %	87		55 %	11	
3	99 %	81		98 %	50		100 %	31	
4	64 %	133		65 %	122		55 %	11	
*Baseline characteristics*									
Age (years)	1.008	442	0.387	1.005	358	0.682	1.023	84	0.272
Gender			0.474			0.46			0.872
Male	67 %	318		67 %	261		68 %	57	
Female	64 %	124		63 %	97		67 %	27	
SBP (mmHg)	1.007	442	0.188	1.003	358	0.598	1.022	84	0.074
DBP (mmHg)	0.998	442	0.815	0.997	358	0.767	1.001	84	0.968
BMI (kg/m ^2^)	1.038	442	0.128	1.049	358	0.08	0.994	84	0.912
TIMI score^a^			0.085						0.085
1	71 %	7					71 %	7	
2	80 %	15					80 %	15	
3	48 %	25					48 %	25	
4	83 %	23					83 %	23	
5	60 %	10					60 %	10	
6 + 7	100 %	3					100 %	3	
NYHA			***p*** **< 0.01**			**0.036**			**0.011**
NYHA I	62 %	324		63 %	260		59 %	64	
NYHA II	71 %	79		69 %	73		100 %	6	
NYHA III & IV	90 %	39		88 %	25		93 %	14	
Number of diseased vessels			***p*** **< 0.01**			***p*** **< 0.01**			0.102
1	58 %	196		58 %	166		57 %	30	
> 1	73 %	246		72 %	192		74 %	54	
*Cardiac history*									
Previous heart failure			0.981			0.836			0.489
Yes	67 %	9		63 %	8		100 %	1	
No	66 %	433		66 %	350		67 %	83	
Previous MI			0.077			0.090			0.536
Yes	72 %	137		72 %	118		74 %	19	
No	64 %	305		63 %	240		66 %	65	
Previous PTCA			***p*** **< 0.01**			***p*** **< 0.01**			0.271
Yes	76 %	148		76 %	126		77 %	22	
No	61 %	294		60 %	232		65 %	62	
Previous CABG			0.541			0.736			0.433
Yes	61 %	31		63 %	27		50 %	4	
No	67 %	411		66 %	331		69 %	80	
*Non-cardiac history*									
CVA/TIA			0.748			0.682			0.924
Yes	69 %	35		70 %	23		67 %	12	
No	66 %	407		66 %	335		68 %	72	
Pulmonary disease			0.103			0.142			0.445
Yes	56 %	47		55 %	35		58 %	12	
No	68 %	395		36 %	323		69 %	72	
PVD			0.086			0.124			0.445
Yes	56 %	57		56 %	45		58 %	12	
No	68 %	385		67 %	313		69 %	72	
Renal failure			0.059			0.126			0.144
Yes	43 %	14		46 %	13		0 %	1	
No	67 %	428		67 %	345		69 %	83	
*Risk factors*									
Diabetes mellitus			***p*** **< 0.001**			***p*** **< 0.001**			**0.009**
Yes	93 %	95		92 %	78		94 %	17	
No	59 %	347		59 %	280		61 %	67	
Hypertension			0.306			0.498			0.324
Yes	68 %	290		67 %	240		72 %	50	
No	63 %	152		63 %	118		62 %	34	
Hyperlipidaemia			0.943			0.427			0.144
Yes	66 %	302		65 %	252		74 %	50	
No	67 %	140		69 %	106		59 %	34	
Current smokers			**0.037**			0.066			0.246
Yes	57 %	85		55 %	58		59 %	27	
No	69 %	357		68 %	300		72 %	57	
Pack years^b^	1.001	442	0.898	1.000	358	0.974	1.002	84	0.877

*BMI* body mass index, *CABG* coronary artery bypass graft, *CAD* coronary artery disease, *CVA* cerebrovascular accident, *DBP* diastolic blood pressure, *MI* myocardial infarction, *NA* not applicable, *NSTEMI* non ST elevation myocardial infarction, *NYHA* New York heart association, *PTCA* percutaneous transluminal coronary angioplasty, *PVD* peripheral vessel disease, *SBP* systolic blood pressure, *TIA* transient ischaemic attack, *TIMI* thrombolysis in myocardial infarction.

^a^only for unstable angina and NSTEMI.

^b^Number of packs per day multiplied with years of smoking.

All multivariate analyses (Table 3) showed that patients with diabetes had a significantly higher chance of receiving DES. The use of DES versus BMS in the stable CAD population was not only associated with diabetes but also with the treating hospital, smoking status, and previous PCI. Patients treated in hospital II or III, patients having diabetes, and patients with a previous PCI had a higher chance of being treated with DES. Patients treated in hospital I and patients who were current smokers had a lower chance of being treated with DES.

**Table 3:** Multivariate analyses therapeutic decision (BMS vs DES)

	Bivariate analyses (OR)	Multivariate analyses (OR)	*p* value*
**Total population (** ***n*** **= 442)**			
*Number of diseased vessels*			
1	0.520	0.560	0.006
> 1	Ref		
*NYHA class*			
NYHA class I	Ref		
NYHA class II	1.478		
NYHA class III + IV	5.311		
*Hospital*			
1	0.565		
2	1.016		
3	45.176		
4	Ref		
Diabetes (yes vs no)	8.680	8.318	p < 0.001
Renal artery disease (yes vs no)	0.368		
Current smoker (yes vs no)	0.599		
Previous MI (yes vs no)	1.486		
PVD (yes vs no)	1.017		
Previous PTCA (yes vs no)	2.045		
*TIMI score* ^a^			
1	Ref		
2	1.20		
3	0.37		
4	2.000		
5	0.600		
6 + 7	646189937		
*Constant*		1.911	p < 0.001
Nagelkerke R^2^		16 %	
**Stable CAD** (***n*** ** = 358**)	**Bivariate analyses (OR)**	**Multivariate analyses (OR)**	***p*** value*
BMI (kg/m ^2^)	1.049		
*Hospital*			
1	0.578	0.466	0.013
2	1.034	1.047	0.884
3	26.671	29.381	0.001
4	Ref	Ref	
Previous MI (yes vs no)	1.513		
*NYHA class*			
NYHA class I	Ref		
NYHA class II	1.280		
NYHA class III + IV	4.319		
*Number of diseased vessels*			
1	0.536		
> 1	Ref		
Current smoker (yes vs no)	0.588	0.404	0.014
Diabetes (yes vs no)	8.454	12.001	p < 0.001
Previous PTCA (yes vs no)	2.103	2.284	0.003
Constant		1.207	0.397
Nagelkerke R^2^		33 %	
**Unstable CAD** (***n*** **= 84**)	**Bivariate analyses (OR)**	**Multivariate analyses (OR)**	***p*** value*
*Hospital*			
1	0.686		
2	1.000		
3	1346229036		
4	Ref		
*NYHA class*			
NYHA class I	Ref		
NYHA class II	1105324892		
NYHA class III + IV	8.895		
Diabetes (yes vs no)	10.146	10.146	0.029
*TIMI score* ^a^			
1	Ref		
2	1.600		
3	0.369		
4	1.900		
5	0.600		
6 + 7	646189937		
SBP (mmHg)	1.007		
*Constant*		1.577	0.069
*Nagelkerke R* ^*2*^		13 %	

*SBP* systolic blood pressure, *BMI* body mass index, *NYHA* New York heart association, *PTCA* percutaneous transluminal coronary angioplasty, *TIMI* Thrombolysis In Myocardial Infarction.

^a^P value of multivariate analyses.

*p = 0.05

## Discussion

This study explored the factors associated with stent choice for Dutch patients diagnosed with stable or unstable CAD. Various factors are associated with the frequency of DES use, including diabetes, previous PCI, number of diseased vessels, NYHA class, smoking and the treating hospital.

Patients requiring a PCI were in most cases treated with at least one DES (66 %), which is in line with the guidelines that suggest that patients with stable CAD should receive a DES if there is no contraindication of prolonged dual antiplatelet therapy [8]. Furthermore, DES is recommended over BMS in NSTEMI or unstable angina patients with diabetes [9]. Since patients with diabetes have a higher restenosis risk than patients without diabetes, DES is considered the most optimal treatment for these patients since DES reduces restenosis compared with BMS. Consequently, diabetes was significantly associated with stent choice in this study. Patients who have been treated before with a PCI were also more likely to receive DES (76 %); these patients have a higher risk of developing restenosis and thus DES was preferred. Patients with multi-vessel disease (73 % DES) and patients with a high NYHA class (range I–IV: 62–90 % DES) were significantly more frequently treated with DES. Studies suggest that patients with multi-vessel disease should be treated with CABG or PCI using DES since these interventions have shown to be more effective than BMS [10]. Patients currently smoking were less often treated with DES.

These clinical factors can be considered as legitimate leading to variation in stent choice. However, 19 % of the variation in stent choice was explained by these factors in the stable CAD population. Beside clinical factors, other potential reasons for treatment variation could exist due to: (1) the operator, (2) the availability and supply of resources, or (3) patient preferences. Considering operator variation, physicians use different methods to decide which stent is most suited for a particular patient. It is known that some physicians are believers of DES and some do not believe in the added value of DES compared with BMS, while BMS is less expensive. In several randomised clinical trials, DES has shown to be more effective than BMS for several indications (e.g. diabetes, long lesions). Some operators strictly follow the results of these trials and the guidelines while other operators also use DES for other indications with a high restenosis risk since guidelines do not provide recommendations concerning the most optimal stent for every type of patient, although it is probably unrealistic to expect this. In our study, one hospital treated almost all patients with DES (99 %); probably DES was used also for ‘off-label’ indications. A Dutch report concluded that world-wide DES is used off-label in 47–81 % of the patients, leading to differences in safety and clinical effectiveness [11]. The second potential reason, availability and supply of resources, focuses on the hospital level. In our analyses, the treating hospital was significantly associated with stent choice even after correcting for clinical factors in the stable CAD group. After adding treating hospital to the regression analysis 33 % of the variation in stent choice could be explained. The analyses showed that the frequency of DES use ranged from 50–99 % of all patients across hospitals. This difference could result from a difference in patient case mix, despite the adjustment for many individual patient characteristics in the analyses. Furthermore, payment arrangements with stent manufacturers and budget constraints may have influenced the stent choice. Another potential reason, patient preference, could have influenced the variation in stent choice. However, we expect this to be minimal since both interventions can be considered to be equally invasive.

### Implications

In general, patients receive the most optimal stent given their clinical characteristics. However, stent choice is also determined by the treating hospital, probably due to operator variation and availability and supply of resources. Variation should only occur due to demographic and angiographic factors. When variation is due to factors other than demographics or angiography findings it could lead to less optimal stent choices and subsequently differences in long-term outcomes.

Patients receiving DES have a lower risk of target lesion revascularisation than patients treated with BMS [3]. However, there is some concern of late stent thrombosis that may occur more frequently after DES than BMS [3]. Besides the implications of treatment variation on the effectiveness, it is also important to consider the costs. While BMS is less expensive than DES, BMS leads to more reinterventions than DES. Several studies have estimated the cost-effectiveness of DES versus BMS and many of these studies concluded that initial DES treatment was overall more expensive than the BMS strategy [12–23]; the reduction in reinterventions did not offset the initial higher stent costs. In most of the studies DES was slightly more effective [12–23] often leading to an incremental cost-effectiveness ratio that could not be considered cost-effective [13, 14, 17, 18, 23]. However, some specific subgroups (diabetes, complex lesions, complex vessels, multi-vessel disease, or a combination of these risk factors) were identified in which DES resulted in a higher health gain in terms of quality-adjusted life-years compared with subgroups that were not at high risk of restenosis and complications. Consequently, in these subgroups, DES was considered more cost-effective. In our study some of these specific subgroups were also associated with a more frequent use of DES.

### Limitations

The factors examined in the analyses explained 13–33 % of the variation in treatment decisions. While the treating hospital was associated with stent choice, it is possible that hospital is a proxy for a pre-existing patient case mix. Many clinical factors were included in the regression models but it is possible that factors that are of predictive value were not included. Furthermore, the underlying reason why the treating hospital is associated with stent choice is unknown. This could be due to the operator (e.g. experience), for which data were not available for our analyses, or the availability and supply of resources might explain the association with the treating hospital, even though the Netherlands has a centrally publicly funded healthcare system.

We were not able to compare patients treated solely with BMS and patients treated solely with DES. Stent choice for DES was defined as a PCI with at least one DES which includes patients treated with only DES but also patients treated with DES in combination with BMS, drug-eluting balloon angioplasty or standard balloon angioplasty. Consequently, the associations that we have found actually explain why some patients receive DES and why other patients did not receive DES.

In addition, this study did not take into consideration the differences in stent choice (different types of DES) despite variation in their effectiveness. For example, the newer ultra-thin strut BMS leads to less restenosis than the thicker strut BMS; a study using the SOLSTICE registry showed that ultra-thin strut BMS leads to low 6-month major adverse cardiac event rates (5.8 %), including target lesion revascularisations [24]. Furthermore, we made no distinction between the types of drug coating (e.g. paclitaxel, sirolimus, or everolimus) used for DES, even though this may affect clinical outcomes.

Lastly, the latest guideline on myocardial revascularisation [25] concluded that the newer generation DES have improved safety outcomes including death, MI and stent thrombosis compared with early-generation DES and BMS. During this study, this guideline was not available and thus it is possible that stent choice might have been somewhat different if the new guidelines had been applicable; DES could be more frequently used. Furthermore, we did not focus on fully bioresorbable stents, which have promising clinical outcomes since they provide desirable transient vessel support without compromising the restoration of normal vessel biology, vessel imaging or treatment options in the long run [26]. Consequently, the stents evaluated in the Circulating Cells cohort may not reflect the stent choices that will be made in the near future.

### Recommendations

This study showed the existence of treatment variation across hospitals that may have an impact on long-term outcomes. It would be interesting to investigate if the treatment variation seen in this cohort will actually lead to differences in long-term outcomes and costs, which could be achieved by increasing the follow-up period. Van der Sijde et al. [27] have also emphasised the role of clinical observations to determine the most appropriate indication for specific types of stents.

## Conclusions

This study showed that several clinical factors were associated with stent choice (DES or BMS) for CAD treatment, including diabetes, smoking, NYHA class, multi-vessel disease and previous PCI. In general, it appears that patients receive the most optimal stent given their clinical characteristics. After correcting for the clinical factors, stent choice was also associated with the treating hospital probably due to operator variation and the availability and supply of specific stent types. These differences may lead differences in long term outcomes.

### Financial support

This work was supported by unrestricted grants from the Center for Translational Molecular Medicine (CTMM), Circulating Cells project (grant 01C-102) and the Dutch Heart Foundation.
